# HRGAN: A Generative Adversarial Network Producing Higher-Resolution Images than Training Sets

**DOI:** 10.3390/s22041435

**Published:** 2022-02-13

**Authors:** Minyoung Park, Minhyeok Lee, Sungwook Yu

**Affiliations:** School of Electrical and Electronics Engineering, Chung-Ang University, 84 Heukseok-ro, Dongjak-gu, Seoul 06974, Korea; crash879@cau.ac.kr

**Keywords:** generative adversarial network, image generation, Inception score, image resolution

## Abstract

The generative adversarial network (GAN) has demonstrated superb performance in generating synthetic images in recent studies. However, in the conventional framework of GAN, the maximum resolution of generated images is limited to the resolution of real images that are used as the training set. In this paper, in order to address this limitation, we propose a novel GAN framework using a pre-trained network called evaluator. The proposed model, higher resolution GAN (HRGAN), employs additional up-sampling convolutional layers to generate higher resolution. Then, using the evaluator, an additional target for the training of the generator is introduced to calibrate the generated images to have realistic features. In experiments with the CIFAR-10 and CIFAR-100 datasets, HRGAN successfully generates images of 64 × 64 and 128 × 128 resolutions, while the training sets consist of images of 32 × 32 resolution. In addition, HRGAN outperforms other existing models in terms of the Inception score, one of the conventional methods to evaluate GANs. For instance, in the experiment with CIFAR-10, a HRGAN generating 128 × 128 resolution demonstrates an Inception score of 12.32, outperforming an existing model by 28.6%. Thus, the proposed HRGAN demonstrates the possibility of generating higher resolution than training images.

## 1. Introduction

While various deep learning algorithms have been extensively studied in recent years, the generative adversarial network (GAN) [1] is one of the most rapidly improved models among many deep learning algorithms. By using an innovative learning process motivated by game theory, GAN can learn a given sample space and generate synthetic samples that mimic features in the trained sample space. Such success of the novel training process of GAN enables us to introduce deep learning algorithms for a new artificial-intelligence task, i.e., realistic sample generation.

The architecture of the original GAN is composed of two deep learning modules, called generator and discriminator. The generator uses lower-dimensional inputs indicating the feature distribution of a dataset, which is generally represented with Gaussian distributions and produces higher-dimensional outputs that correspond to synthetic samples. The discriminator learns features of a real dataset by attempting classification between the synthetic samples and real samples. Meanwhile, the generator also learns the features by deceiving the discriminator. The parameter weights of the generator are optimized with an inverse target to the discriminator. Thus, it can be interpreted that these two modules play a game to detect and deceive each other. The adversarial and competitive training process between the two modules is one of the main characteristics of GAN.

After the training, the generator can produce as many realistic but synthetic samples as desired since such a training process induces the features to be mapped onto the noise variables, which are the inputs of the generator. Therefore, using the Monte Carlo sampling method on the noise variables, a set of synthetic samples can be made, which eventually corresponds to a deep-learning-based sample generation. Such a new sample generation framework has been studied extensively, and conventionally, has been applied to image datasets to make realistic images.

However, there is a critical limitation in the original GAN framework in that the generator can produce the same resolution samples as the original samples. For example, if a generator and a discriminator are trained with the CIFAR-10 image dataset, which consists of 32 × 32 resolution images, the trained generator can produce only 32 × 32 resolution images as well. Hence, since it has been found that the quality and degree of recognition of generated image samples are related to a higher resolution of the images, such a limitation significantly reduces the quality of the generated images.

In order to synthesize high-resolution images by GAN, a modified GAN model called super resolution GAN (SRGAN) [2] has been studied. However, SRGAN also has two limitations: First, SRGAN cannot be used for the sample generation task since the model takes a lower resolution image as an input. Therefore, distinct from the original GAN that takes noise variables as its inputs, SRGAN is not a generative model. Second, for the training process of SRGAN, higher resolution samples are still required. For instance, when SRGAN aims to synthesize 128 × 128 resolution images from 64×64 resolution input images, a training set of 128 × 128 resolution images is required for its training process. Hence, SRGAN cannot synthesize 128 × 128 resolution images if a lower resolution ground-truth training set is given, such as 32 × 32 resolution images in the CIFAR-10 dataset. Moreover, other models for image super resolution [3] take low-resolution images as inputs, which does not correspond to image generation and requires high-resolution images for the training.

In this paper, we introduce a GAN model for the higher resolution sample generation without using higher resolution training samples. By using the proposed model, higher resolution image samples can be generated even though the same resolution real samples do not exist. For example, while the CIFAR-10 dataset consists of only 32 × 32 resolution images, higher resolution images, such as 64 × 64 and 128 × 128 resolution images of the CIFAR-10, can be produced by the proposed model, where the real images of those resolutions do not exist.

Compared to the original GAN, the proposed model employs an additional deep learning classifier, which is pre-trained and conventionally used. By the modification, the proposed model aims at learning conventional and general features in various objects in the real world. Therefore, the generated higher resolution images become more realistic from the learning with the pre-trained classifier. Such a modification is motivated by score-guided GAN (ScoreGAN) [4], in which it has been verified that the metric to evaluate GAN can also be used for the training of the generator. We leverage the advantage of using a pre-trained classifier and introduce it for the higher resolution sample generation with GAN.

## 2. Methods

### 2.1. Generative Adversarial Networks

A GAN model consists of two neural network structures, a generator and a discriminator. Since features of a dataset can be represented as a lower-dimensional distribution than the sample distribution, the generator aims at representing the data samples to be feature distributions by mapping them onto inputs of the generator. Therefore, the generator becomes possible after the GAN training process, with a noise vector that indicates a feature vector.

In order to make the generator learn the sample distribution, the discriminator is employed in the training process. In the training, the discriminator takes produced samples by the generator and real samples in the dataset. Then, the discriminator is trained to be a classifier that differentiates between the produced samples and real samples. During the training of the discriminator, the generator is also trained by deceiving the discriminator, resulting in learning the features of the dataset. Hence, the GAN training can be likened to a game between the generator and the discriminator.

Such a training process can be represented as target functions as follows:

(1) $$\underset{D}{\max}\;\underset{G}{\min}V\left(G,\;D\right)=\underset{x\sim q_{data}\left(x\right)}{E}\left[ℒ\left(D\left(x\right);\theta \right)\right]+\underset{z\sim p\left(z\right)}{E}\left[ℒ\left(D\left(G\left(z\right)\right);\theta \right)\right],$$

where G and D represent the generator and the discriminator, respectively, x is a set of real samples, z is a set of noise vectors, ℒ is a specific loss function, and θ indicates a set of parameters of the loss function.

After the training, synthetic samples can be generated by G(z) where z~p(z), i.e., using random feature vectors as the inputs of the generator. Due to the outstanding performance to learn sample spaces and produce synthetic samples, these GAN models have been commonly used for image datasets to generate synthetic images, which had been regarded as a complex problem.

### 2.2. Generation of Higher Resolution Images

In general, resolution is one of the most important factors in image quality because high-resolution images have more details than low-resolution ones. Such a factor as image data has been addressed in [5]. Therefore, compared to low-resolution images, it has been verified that high-resolution images have a better probability that they are classified as correct labels of the corresponding images, which demonstrates superb recognizability of high-resolution. Hence, it is important to be able to generate high-resolution images to obtain a better quality of the images.

However, the produced images of the most generative models have the same resolution as the real images that are employed for the training. In Equation (1), such a factor can be interpreted that the dimension of x and G(z) should be the same. Since the input dimension of the discriminator must be the same for real images and produced images, it is natural that the produced images have a limitation in that their resolution cannot exceed the resolution of real images. Such a limitation becomes critical when high-resolution images cannot be obtained. For example, the CIFAR-10 dataset is one of the conventional datasets to evaluate deep learning models, including GAN models [1,6,7]. However, the resolution of the CIFAR-10 is 32 × 32, which means that the ordinary GAN cannot produce high-resolution images due to the limitation in the dataset.

To handle this limitation, this paper aims to propose a GAN model, called higher resolution GAN (HRGAN), that produces higher resolution images that exceed the resolution of original images. In HRGAN, a pre-trained classifier with a score is introduced to evaluate the produced higher resolution images while they are still evaluated by the discriminator. However, since the resolution of the produced images and original images are different, a down-sampling process is employed for the produced images. This training process of HRGAN can be interpreted as follows: the main features in a dataset are learned by the discriminator, whereas fine-grained features in the higher resolution, such as edges of objects, are trained by the pre-trained classifier.

**Figure 1:**
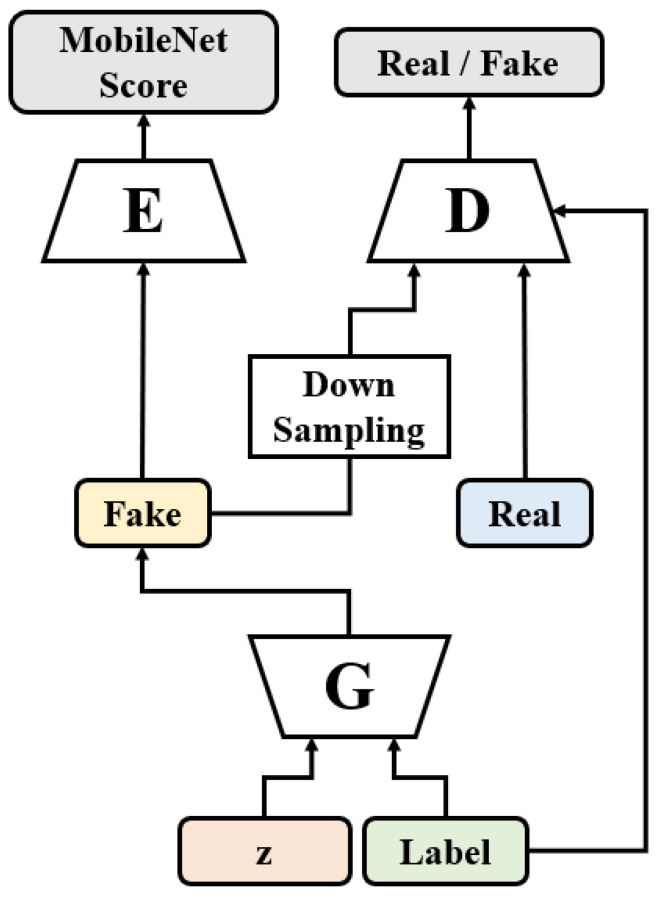
The proposed HRGAN model. The ***z*** is a random latent vector sampled from a normal distribution, **Label** is a class vector expressed by an one-hot vector, X indicates training images, and therefore, G(***z***, **Label**) becomes synthetic images.

Figure 1 shows the architecture of HRGAN, where G, D, and E represent the generator, discriminator, and evaluator, respectively. The synthetic images, i.e., G(***z***, **Label**) in the figure, have a higher resolution than the real ones, by the up-sampling layers in the generator. For example, when the CIFAR10 (or CIFAR100) dataset is used, the size of the real images is 32 × 32, whereas the size of the fake images can be 64 × 64, 128 × 128, or even larger, according to the number of up-sampling layers in the generator. The discriminator in HRGAN is the same as the ordinary GAN [1,7], so it expects an image that has the same resolution as the real training images. Thus, a down-sampling process is adopted between the generator and the discriminator.

One of the main ideas of HRGAN is the use of the evaluator, which is represented as E in Figure 1. The evaluator computes an evaluation score of generated images. Then, the score is backpropagated to the generator to maximize the score. This training process with the evaluator will be explained further in Section 2.4. There are several metrics that can be used for the score of the evaluator to assess generated images, but it should be noted that it is not straightforward since generative models synthesize the images without any ground truth images. Thus, the most popular metrics to access the image quality, such as peak signal-to-noise ratio (PSNR) and structural similarity index measure (SSIM) [8], cannot be used in the evaluator because they require the ground truth images. Therefore, the evaluator uses a metric to access GAN models. Currently, the most popular way to evaluate a GAN model is to use the Inception score (IS) [9] or Fréchet Inception distance (FID). Among the two metrics, the evaluator employs the Inception score since FID requires a very high computational cost, especially in the matrix square root computation. Since HRGAN utilizes the score during the training, such a high computational cost of FID is critical to be used in the evaluator.

The general concept of the Inception score is utilized for training HRGAN, but a modified score, called MobileNet [10] score, is used instead of the Inception score. The Inception score is originally obtained by a pre-trained InceptionV3 [11] network. However, since the evaluator is used both in the forward and the backward computation paths during the training process of HRGAN, a heavy model significantly increases the computational complexity, resulting in infeasible computational time. Thus, HRGAN uses a small version of a pre-trained network, called MobileNetV3-small [12], which demonstrates a good tradeoff between the complexity and the performance.

Although it is expected that the Inception network shows better performance as the evaluator of HRGAN, note that the proposed HRGAN is an integrated model consisting of a high-resolution GAN and a classifier, both of which have high complexity. For instance, when HRGAN employs the Inception network instead of the MobileNet, it failed in training due to a memory issue with the GPU, while an NVIDIA RTX A6000 with 48 GB GPU memory was used, which has the most extensive GPU memory among the NVIDIA RTX GPU series. Thus, for a feasible computation in conventional systems, including sensor systems, it is crucial to minimize the complexity of the evaluator.

In addition, note that the InceptionV3 consists of about 23 M parameters whereas MobileNetV2 is composed of about 3 M parameters, while the difference in the top-5 accuracy of the ImageNet dataset is within 5%p [10]. Hence, owing to such complexity and performance, it is more appropriate to use the MobileNet for HRGAN.

**Figure 2:**
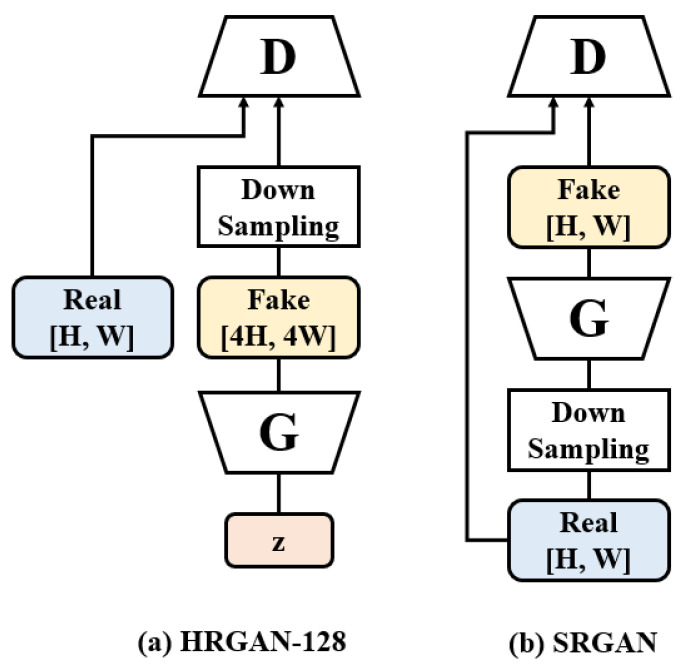
The comparison of the architectures of (**a**) HRGAN and (**b**) SRGAN. When the models are trained with the CIFAR-10 dataset, **H** and **W** become 32, signifying that the maximum resolution of generated images in SRGAN is 32 × 32 while HRGAN can produce 64 × 64 and 128 × 128 resolutions of images.

While HRGAN and the existing SRGAN [2] seem that they have a similar concept in producing higher resolution images, they are completely different from each other in terms of the objective, architecture, and operation process of the models. Figure 2 compares the proposed HRGAN with the SRGAN. More precisely, Figure 2a illustrates the case where 32 × 32 real training images are used to generate 128 × 128 fake images in the proposed HRGAN, whereas Figure 2b illustrates the case where 128 × 128 real images are used to train the generator that converts a 32 × 32 image into a 128 × 128 image in the SRGAN.

Here, one of the main differences is the existence of high-resolution images in the training process. In the proposed HRGAN, low-resolution (e.g., 32 × 32) training images are used to generate high-resolution (e.g., 128 × 128) images, whereas high-resolution (e.g., 128 × 128) images are required in the SRGAN to train the generator. Another important difference is that the input to the HRGAN generator is a latent random vector similar to early GANs [1,6,7], while the input to the SRGAN generator is a down-sampled image and not a random vector. Thus, the objective of HRGAN is generating images by using feature vectors, while the objective of SRGAN is to enhance the resolution of low-resolution images, which can hardly be interpreted as sample generation. In other words, the role of the SRGAN generator is to convert (i.e., up-scale) a low-resolution image to produce a high-resolution (i.e., super resolution) version, whereas the role of the HRGAN generator is to generate a high-resolution image out of a random vector. In addition, the SRGAN cannot be applied when the dataset only contains low-resolution images, e.g., CIFAR-10, since the model requires high-resolution images during its training.

### 2.3. Architecture

#### 2.3.1. Generator

**Figure 3:**
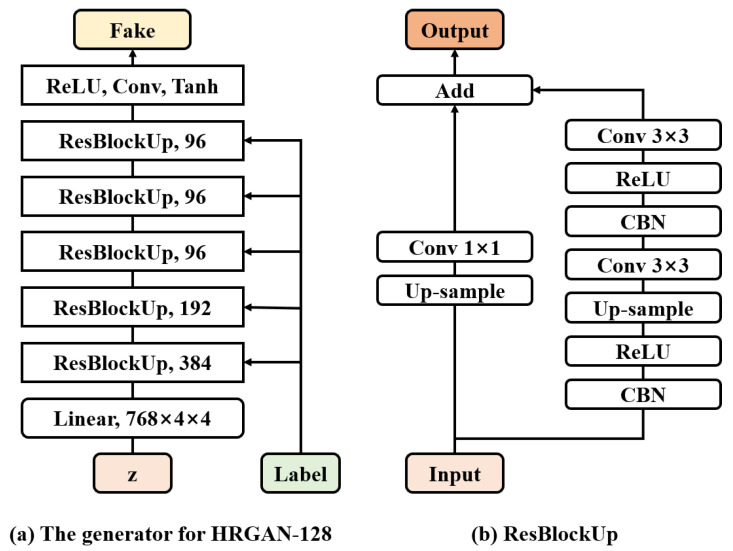
The structures of (**a**) the HRGAN-128 generator and (**b**) the up-sampling residual block.

Figure 3a shows the overall block diagram of the HRGAN-128 generator and Figure 3b shows a more detailed structure of the up-sampling residual block (ResBlockUp) [13,14,15], which is the basic building block in the generator. In Figure 3a, the HRGAN-128 generator contains several up-sampling residual blocks that are used to increase the size of the input images or input feature maps. The number shown at the end of each up-sampling residual block represents the channel size. Note that the HRGAN-128 generator contains five up-sampling residual blocks, whereas the HRGAN-64 generator contains four up-sampling residual blocks. In this manner, the resolution of generated images can be determined by the number of up-sampling residual blocks of the generator of HRGAN.

Each up-sampling residual block contains the main path and a shortcut path, as shown in Figure 3b. Among the two paths, the main path consists of conditional batch normalization (CBN) [14,15,16,17,18], ReLU activation, up-sampling, and vanilla convolution layers. As mentioned in Section 2, the proposed method uses the CBN to feed the label information to the generator. The up-sampling layer uses the nearest neighbor method, and it doubles the resolution of the input features. The 3 × 3 convolution layers perform a convolution with a unit stride so that the feature map size does not change after the operation. The shortcut path also contains an up-sampling layer to make the size of the output the same as that of the main path output. It also contains a 1 × 1 convolution layer to change the channel size in a flexible way.

#### 2.3.2. Discriminator

**Figure 4:**
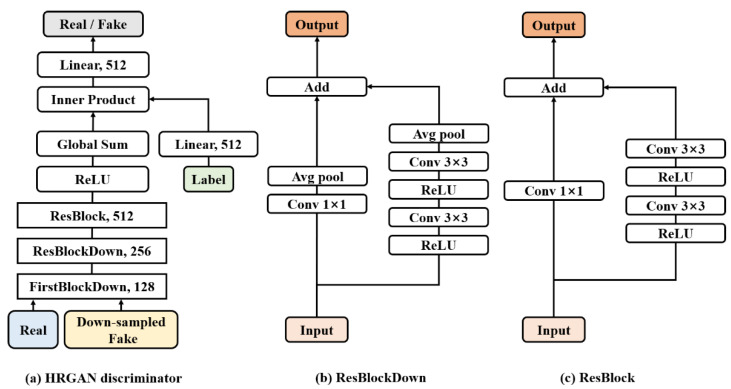
The structures of (**a**) the HRGAN discriminator, (**b**) the down-sampling residual block, and (**c**) the residual block without sampling.

The architecture of the discriminator is basically the same as the ordinary conditional GAN (cGAN) [6,14,15]. Figure 4a shows the block diagram of the HRGAN discriminator, and Figure 4b,c show the two main components, which are the down-sampling residual block (ResBlockDown) [13,14,15] and the residual block (ResBlock) [13,14,15]. Note that the HRGAN discriminator expects a 32 × 32 size input image, regardless of the size of the generated fake image, and thus, the same structure of discriminator in Figure 4a can be used for both HRGAN-64 and HRGAN-128. Two down-sampling residual blocks are used in the discriminator to make the feature map size to be 8 × 8. The inner product block is required to feed the label information to the discriminator by using the projection method in [15].

Both the down-sampling residual block and the residual block contain the main path and a skipped path [14,15]. The only difference is that the down-sampling residual block includes average pooling layers to reduce the feature map size in half. The skip paths of both blocks include 1 × 1 convolution layers for adjusting channel size. Notice that the first down-sampling residual block in Figure 4a does not contain the first ReLU layer in Figure 4c because the input to this block is not a feature map but an input image.

### 2.4. Objective Functions

The following equation shows the discriminator loss function of the proposed method:

(2) $$\max V_{D}\left(G,\;D\right)=\underset{x\sim q_{data}\left(x\right)}{E}\left[\min\left(0,-K+D\left(x,y\right)\right)\right]+\underset{z\sim p\left(z\right)}{E}\left[\min\left(0,-K-D\left(G\left(z,y\right),y\right)\right)\right],\;$$

where D and G refer to the discriminator and the generator, and the data ***x***, ***y***, and *z* refer to a real input image, one-hot label vector, and a random latent vector that is randomly sampled from a normal distribution, respectively. The Lipschitz constant *K* is set to 1.0 for training, following WGAN [19] where the concept of Lipschitz continuous in GAN was proposed, but any other positive real numbers can be used. To effectively train the model, the proposed method adopts the hinge loss [20], which updates weights only when the outputs of the discriminator are informative. Although the Lipschitz constant of one has been employed in the original hinge loss, it is generalized in this study with Equation (2). While the regularization methods for GANs commonly aim at maintaining the Lipschitz continuity in GAN with the Lipschitz constant of one, the relationships between the value of Lipschitz constant and performance should be further investigated. Therefore, in this study, Equation (2) with a generalized hinge loss for the GAN training is proposed for such possibilities of further research.

While the discriminator loss function contains only the adversarial loss term, the generator loss function of the proposed method contains two terms (i.e., the adversarial loss component and the MobileNet score loss component) as follows:

(3) $$\min V_{G}\left(G,\;D,\;E\right)=-\underset{z\sim p\left(z\right)}{E}\left[D\left(G\left(z,y\right)\right)\right]+\lambda_{MS}L_{MS}\left(G,\;E\right).\;$$

In Equation (3), the network E refers to the evaluator, and the coefficient *λ_MS_* is a controllable parameter that is used to decide the relative weights of the adversarial loss term and the MobileNet score loss term. The adversarial loss term, i.e., the first term in Equation (3), is the same as the loss function used in the WGAN [19]. On the other hand, the MobileNet score loss, i.e., the second term in Equation (3), is defined as follows:

(4) $$L_{MS}=\max\left(0,\log\left(\frac{MS_{real}\;\cdot \;\sqrt{HR}}{MS_{fake}}\right)\right).$$

Here, the coefficient *HR* indicates the ratio of the size of the generated images to that of the real images. For example, if the resolution of the generated images is 64 × 64 or 128 × 128 when the resolution of the training images is 32 × 32, then the coefficient *HR* is set to 2 and 4, respectively. Such a term allows the model not to overfit the evaluator by adopting a maximum score that the model can achieve. In Equation (4), *MS* refers to the MobileNet score, which is defined as follows:

(5) $$MS=\exp(D_{KL}(p\left(y|x\right)||p\left(y\right)))=\exp\left(\frac{1}{\left|x\right|}\underset{x\in x}{\sum }\underset{y\in Y}{\sum }p\left(y|x\right)\log\frac{p\left(y|x\right)}{p\left(y\right)}\right).\;$$

where *D_KL_* represents the *KL* divergence, *X* refers to the set of all generated images, and *Y* refers to the set of all classes. As mentioned in Section 2.2, the MobileNet score uses the MobileNetV3 [12] network, whereas the original Inception score uses the Inception V3 [11] network. In Equation (4), $MS_{real}$ is MobileNet score from the real dataset, while $MS_{fake}$ is the one from the generated images.

It should be noted that $MS_{real}$ is computed (i.e., pre-computed) before the HRGAN training process. That is, before we train the proposed HRGAN, we apply the real dataset to the evaluator, and compute $MS_{real}$ by using Equation (5). Then, when we train the HRGAN, we apply the fake dataset (as shown in Figure 1) and compute $MS_{fake}$ by using Equation (5) once again. Thus, $MS_{real}$ in Equation (4) is essentially a constant when we train the HRGAN. It should also be noted that the sample space for $MS_{real}$ is the whole real dataset, whereas the sample space for $MS_{fake}$ is one mini batch of the generated samples.

It is important to note that the MobileNet score of the real dataset is necessary for the proposed method. However, according to the paper [21] addressing the Inception score, an image with a high score does not always guarantee a better quality of images. Therefore, it is critical to maintain a balance between the two objectives of the generator in order not to overfit the evaluator.

Thus, a maximum is used for $L_{MS}$ in the proposed method to avoid that the MobileNet score of generated images exceeds those for the real ones, and $MS_{real}$ serves as the maximum value. In other words, the proposed method aims to maintain the MobileNet score of generated images as similar as possible to that of real images.

### 2.5. Training

For both the generator and the discriminator, the Adam optimizer [22] is used. The proposed HRGAN also adopts the spectral normalization method [23] for stable training. The learning rates for the generator optimizer and the discriminator optimizer are set to 1.0 × 10^−4^ and 2.0 × 10^−4^ [10], respectively. The hyperparameters (β1, β2) that we used for Adam optimizers are (0.0, 0.99). While both the real and generated images are required in training the discriminator, only the generated images are required in training the generator. As a result, the batch size for the generator is set to 128, while the batch size for the discriminator is set to 64. The total number of iterations for the parameter updates in both networks is set to 10^5^. For every single iteration during training, the generator updates parameters to minimize the objective function consisting of the adversarial loss and the MobileNet score loss. More detailed training algorithms are shown in Algorithm 1.

**Algorithm 1** HRGAN training algorithm.**Model**: *D*: discriminator. *G*: generator. *G_d_*: generator down-sampling. *E*: pre-trained MobileNetV3-small. **Parameter**: *θ_disc_*: discriminator parameters. *θ_gen_*: generator parameters. **Input**: *x*: data set. *y*: one-hot encoded label vector. *z*: random noises sampled from a normal distribution. *w*: one-hot encoded label vector converted from random integer sampled from a normal distribution.
**Require**: *α*: the learning rate of the generator. *m*: discriminator batch size. *n*: the ratio of discriminator and generator backpropagation. *cls*: the number of classes. *MS_real_*: the pre-calculated MobileNet score of real data set. *HR*: the ratio of high-resolution output images and real images. $1:\;\mathrm{Initialization}\;\;\theta_{disc},\theta_{gen}\;\leftarrow \;\mathrm{Xavier}\;\mathrm{uniform}$ $2:\;\mathrm{while}\;\theta_{gen}\;\mathrm{has}\;\mathrm{not}\;\mathrm{converged}\;\mathrm{do}$ 3:    for i=0,⋯,n do $4:\;\;\;\;\;\;\;\;\mathrm{Sample}\;{\left\{x^{\left(i\right)}\right\}}_{i=1}^{m}\;\sim {\;P}_{r}\;a\;\mathrm{batch}\;\mathrm{of}\;\mathrm{images}\;\mathrm{from}\;\mathrm{the}\;\mathrm{real}\;\mathrm{data}\;\mathrm{set}.$ $5:\;\;\;\;\;\;\;\;\mathrm{Sample}\;{\left\{y^{\left(i\right)}\right\}}_{i=1}^{m}\;\sim {\;P}_{r}\;a\;\mathrm{batch}\;\mathrm{of}\;\mathrm{one}\text{-}\mathrm{hot}\;\mathrm{label}\;\mathrm{vectors}\;\mathrm{from}\;\mathrm{the}\;\mathrm{real}\;\mathrm{data}\;\mathrm{set}.$ $6:\;\;\;\;\;\;\;\;\mathrm{Sample}\;{\left\{z^{\left(i\right)}\right\}}_{i=1}^{m}\;\sim \;p\left(z\right)\;a\;\mathrm{batch}\;\mathrm{from}\;a\;\mathrm{normal}\;\mathrm{distribution}.$ $7:\;\;\;\;\;\;\;\;grad_{\theta_{disc}}\;\leftarrow \;\nabla_{\theta_{disc}}\left[\begin{array}{c} \frac{1}{m}\overset{m}{\underset{i=1}{\sum }}\min\left(0,-k+D\left(x^{\left(i\right)},y^{\left(i\right)}\right)\right)\\ +\frac{1}{m}\overset{m}{\underset{i=1}{\sum }}\min\left(0,-k-D\left(G_{d}\left(z^{\left(i\right)},y^{\left(i\right)}\;\right),y^{\left(i\right)}\right)\right) \end{array}\right]\;$ $8:\;\;\;\;\;\;\;\;\theta_{disc}\leftarrow \theta_{disc}+2\alpha \cdot$$Adam\left(\theta_{disc},grad_{\theta_{disc}}\right)$ 9:    end for $10:\;\;\;\;\mathrm{Sample}\;{\left\{z^{\left(i\right)}\right\}}_{i=1}^{2m}\;\sim \;p\left(z\right)\;a\;\mathrm{batch}\;\mathrm{from}\;a\;\mathrm{normal}\;\mathrm{distribution}.$ $11:\;\;\;\;\mathrm{Sample}\;{\left\{w^{\left(i\right)}\right\}}_{i=1}^{2m}\;\sim \;U\left(0,\;cls-1\right)\in z\;a\;\mathrm{batch}\;\mathrm{of}\;\mathrm{one}\text{-}\mathrm{hot}\;\mathrm{label}\;\mathrm{vectors}\;\mathrm{from}\;a\;\mathrm{uniform}\;\mathrm{distribution}.$ $12:\;\;\;\;P\left(c|z^{\left(i\right)},w^{\left(i\right)}\right)\leftarrow \;\mathrm{softmax}\left(E\left(G\left(z^{\left(i\right)},w^{\left(i\right)}\right)\right)\right)$ $13:\;\;\;\;P\left(c\right)\;\leftarrow \;\frac{1}{2m}\overset{2m}{\underset{i=1}{\sum }}P\left(c|z^{\left(i\right)},w^{\left(i\right)}\right)$ $14:\;\;\;\;MS_{fake}\;\leftarrow \;\exp\left(\frac{1}{2m}\overset{2m}{\underset{i=1}{\sum }}\overset{cls}{\underset{c=1}{\sum }}P\left(c|z^{\left(i\right)},w^{\left(i\right)}\right)\log\frac{P\left(c|z^{\left(i\right)},w^{\left(i\right)}\right)}{P\left(c\right)}\right)$ $15:\;\;\;\;grad_{\theta_{gen}}\;\leftarrow \;\nabla_{\theta_{gen}}\left[\begin{array}{c} -\frac{1}{2m}\overset{2m}{\underset{i=1}{\sum }}D\left(G_{d}\left(z^{\left(i\right)},y^{\left(i\right)}\;\right),y^{\left(i\right)}\right)\\ +\max\left(0,\log\left(MS_{real}\;\cdot \;\sqrt{HR}\right)-\log MS_{fake}\;\right) \end{array}\right]$ $16:\;\;\;\;\theta_{gen}\leftarrow \theta_{gen}-\alpha \cdot$$Adam\left(\theta_{gen},grad_{\theta_{gen}}\right)$ 17: end while

## 3. Results

We conducted several experiments to evaluate the efficiency of the proposed HRGAN. In the experiments, we used not only the HRGAN-64 and HRGAN-128 networks explained in Section 2.3, but also used the HRGAN-32 network that is designed to synthesize images of the same size as the real images. The HRGAN-32 generator has the same structure as the HRGAN-128 generator in Figure 3a, except that it lacks the last two up-sampling residual blocks.

We trained the HRGAN-32 network in two ways in order to validate the effectiveness of targeting the MobileNet score. Specifically, we compared the HRGAN-32 models with and without the MobileNet score term. The CIFAR10 and CIFAR100 datasets are used in this evaluation. Table 1 shows the Inception scores of the HRGAN-32 models. As shown in the results, targeting the MobileNet score demonstrates performance gains of 0.33 and 0.78 in CIFAR10 and CIFAR100 datasets. Therefore, the effectiveness of the proposed target can be verified since the Inception score is improved. Such an improvement can be achieved by enforcing the generated data and the real data to have similar output features obtained by the evaluator. Since the evaluator was trained with a different large-scale dataset, i.e., the ImageNet dataset, it can also be interpreted that the generated images and the real images have common features in terms of generalized shapes of objects.

In addition, to evaluate higher resolution images produced by HRGANs, we performed experiments with HRGAN-32, HRGAN-64, and HRGAN-128 in terms of the Inception score. As in the previous experiment, we obtained the Inception scores during the training, both with and without the MobileNet score in Equation (5). We also employed the same condition for the experiment since such a comparison can directly provide quantitative performance improvement by the proposed method.

Table 2 shows the Inceptions scores for the HRGANs evaluated with the CIFAR10. As a result, the Inception score increased as higher resolution images were produced. For instance, with the proposed evaluator, HRGAN-64 achieved an Inception score of 10.62, which outperforms HRGAN-32 by 1.85. Such a result demonstrates that the proposed scheme of generating higher resolution images is valid and can produce more recognizable images. In addition, the improvement of the Inception score is more significant when the MobileNet score is used for the training of the generator. This result validates the effectiveness of the proposed method using the evaluator and score in HRGAN.

Although it is possible to use additional up-sampling layers to increase the resolution of generated images, the improvements of the Inception score tend to saturate as up-sampling layers are added. For example, HRGAN-64 outperforms HRGAN-32 by 1.85, but the improvement of HRGAN-128 compared to HRGAN-64 was 1.70. Such a result signifies that there is a form of limitation to generate higher resolution images than a certain critical point while the model successfully produces ×2 and ×4-scaled synthetic images.

Similarly, it is expected that the other parameters in HRGAN can affect the Inception performance. For example, as mentioned in the previous sections, since a better evaluator can enhance the performance of HRGAN, the performance can further increase when superb evaluators are employed while the MobileNet is used in this study owing to a hardware capacity issue. Therefore, it is anticipated that an improved Inception score can be obtained by HRGANs with superb evaluators if conventional and better hardware can be obtained in future research.

In addition, because the Inception score is computed as Equation (5) with the Inception network instead of MobileNet, the saturation of the Inception score concerning the resolution of generated images is natural. The input size of the Inception network is 299 × 299. Therefore, the nearest up-sampling method is commonly used to compute the Inception score and employ generated images as the inputs of the Inception network. In this process, generated images with higher resolution are noticeable because they have more features that can be recognized by the Inception network. However, the Inception score is saturated as the resolution of generated images approaches 299 × 299. Thus, it can be interpreted that this saturation is caused by the limitation of the Inception score.

Table 3 and Table 4 compare HRGANs with existing models in terms of Inception score. The Inception scores with the CIFAR10 and CIFAR100 are compared in Table 3 and Table 4, respectively. As shown in the comparisons, the Inception score of the proposed HRGAN-128 outperformed the other existing models. For example, HRGAN-128 with the CIFAR100 dataset demonstrated an Inception score of 10.90, which outperforms SNGAN [23] by 17.2%. Interestingly, HRGAN-128 with the CIFAR10 produced images having a higher Inception score compared to real images. Such a result also indicates that the proposed method has the potential to obtain superb quality synthetic images by enhancing the resolution of images.

**Figure 5:**
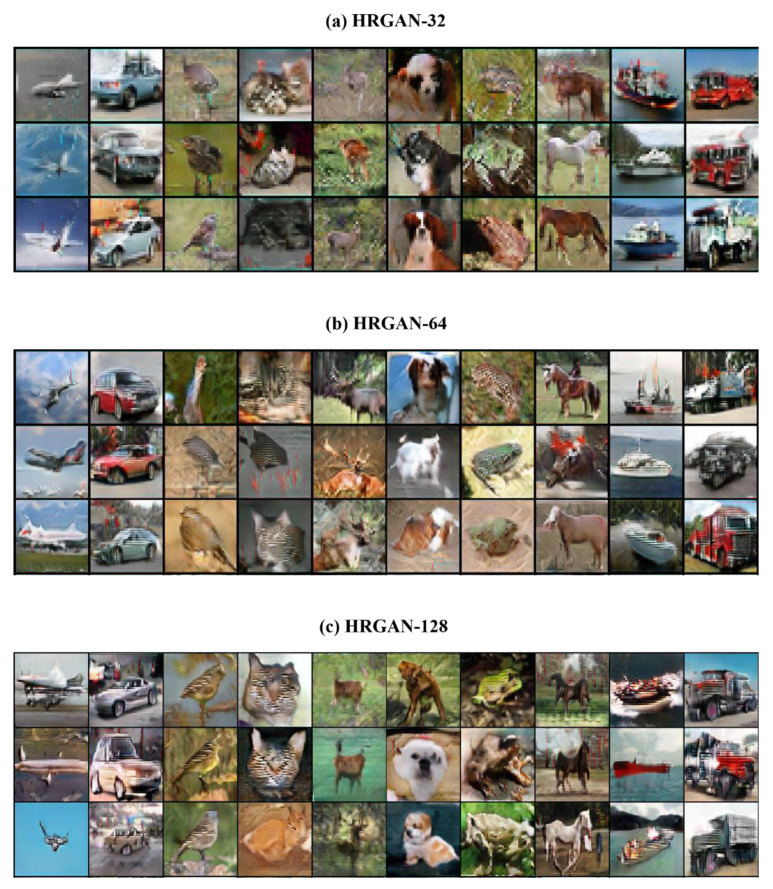
Randomly generated image samples of CIFAR10 by HRGANs with different resolutions.

**Figure A1:**
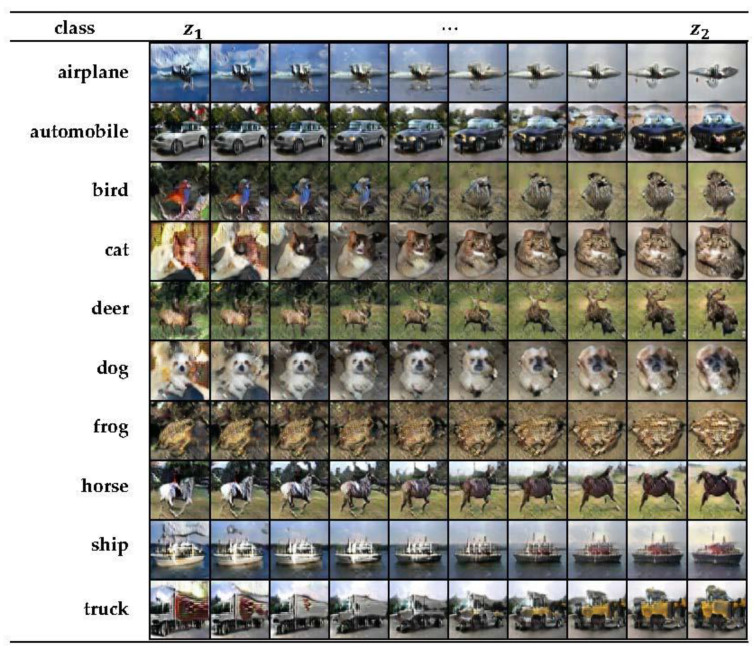
The interpolation images between two latent vectors. This image is generated by HRGAN-128 and down-sampled to the size of 64 × 64.

To compare generated image samples with different resolutions in the view of image quality, randomly generated CIFAR10 images by HRGAN-32, HRGAN-64, and HRGAN-128 are shown in Figure 5. As mentioned in the previous section, the proposed evaluator adjusts fine-grained features of images. As shown in the images, it is obvious that the edges of the objects in HRGAN-64 and HRGAN-128 were sharpened so that the images became more natural, even if the resolutions are higher than the original images. Consequently, it can be interpreted that such a fine-tuning process for generated images by the evaluator enhances the recognizability of the generated higher resolution images. Additionally, to demonstrate the continuity of generated images with respect to the latent space, generated images for interpolated latent vectors are shown in Appendix A (Figure A1).

## 4. Conclusions

While recently introduced GAN models successfully generate synthetic images that have similar features to real images, there has been a constraint in that the maximum resolution of generated images is limited to the same resolution of real images. The proposed HRGAN handles this limitation using a pre-trained network called evaluator and a score for the training of the generator. In the experiments, HRGAN demonstrated the possibility of generating higher resolution images than the original images. In addition, the HRGANs outperformed existing models in terms of the Inception score, resulting in the generation of more recognizable images by increasing the resolution.

Although HRGAN showed a promising result to generate higher resolution images, there are several limitations in the model. First, the performance of the model can be changed and determined by the pre-trained evaluator. We used the MobileNet due to the time complexity of the GAN training, but a better evaluator presents the possibility to further enhance the performance of HRGAN. Second, while the model successfully generates synthetic images that have four times higher resolution than real images, a more enhanced resolution of images can hardly be obtained. These limitations should be investigated further in future work.

## Figures and Tables

**Table 1:** The Inception scores for HRGAN-32 with and without the backpropagation of the proposed score.

Dataset	Model	Inception Score	Improvement
CIFAR10	$w/o\;L_{MS}$	8.44 ± 0.08	0.33
	$\mathrm{with}\;L_{MS}$	8.77 ± 0.09	
CIFAR100	$w/o\;L_{MS}$	8.81 ± 0.12	0.78
	$\mathrm{with}\;L_{MS}$	9.59 ± 0.16	

**Table 2:** The Inception scores with and without the Inception score loss.

Model	$\mathrm{Inception}\;\mathrm{Score}\;\mathrm{without}\;L_{MS}$	$\mathrm{Inception}\;\mathrm{Score}\;\mathrm{with}\;L_{MS}$	Improvement
HRGAN-32	8.44 ± 0.08	8.77 ± 0.09	0.33
HRGAN-64	8.69 ± 0.11	10.62 ± 0.12	1.93
HRGAN-128	-	12.32 ± 0.11	-

**Table 3:** The Inception scores of CIFAR10 dataset. The bold indicates the proposed HRGANs.

Model	Inception Score
Real Images	11.26 ± 0.13
Conditional DCGAN [6]	6.58
AC-WGAN-GP [5]	8.42 ± 0.10
CAGAN [24]	8.61 ± 0.12
Splitting GAN [25]	8.87 ± 0.09
BigGAN [14]	9.22
MHingeGAN [26]	9.58 ± 0.09
**HRGAN-64**	**10.62 ± 0.12**
**HRGAN-128**	**12.32 ± 0.11**

**Table 4:** The Inception scores of CIFAR100 dataset. The bold indicates the proposed HRGANs.

Model	Inception Score
Real Images	14.91 ± 0.20
ControlGAN [27]	9.32 ± 0.11
SNGAN [23]	9.30 ± 0.08
**HRGAN-64**	**10.34 ± 0.11**
**HRGAN-128**	**10.90 ± 0.22**
